# Effect of Sports Therapy Interventions on Severity of Symptoms in the Inpatient Treatment of Depression

**DOI:** 10.1192/j.eurpsy.2025.685

**Published:** 2025-08-26

**Authors:** M. Ziegenbein, M. Wendt, K. Friedrich

**Affiliations:** 1 Psychiatry and Psychotherapy, Wahrendorff Clinic, Sehnde, Germany

## Abstract

**Introduction:**

Physical activity has been identified as a therapeutic intervention for the treatment of depressive disorders. It can be conducted as a standalone intervention or as a complementary therapy method within a multimodal treatment framework. Engaging in physical activity is important for maintaining physical and mental health. There is a need for research on the implementation of exercise therapy interventions that takes into account everyday clinical practice outside the controlled conditions in study protocols. Physical activity is particularly promoted in the context of sports therapy.

**Objectives:**

Therefore, the objective of this study is to examine the impact of exercise therapy in a day clinic setting on depressive symptom severity and cardiovascular fitness.

**Methods:**

The study sample includes patients with a primary diagnosis of depression who had completed a minimum of four and a maximum of nine weeks of inpatient treatment in a psychotherapeutic and psychosomatic hospital in Germany. Patients in the intervention group (IG) receive exercise therapy at least twice a week throughout their treatment, while patients in the control group received a maximum of 0.5 units/week of exercise therapy. As part of the multimodal treatment setting, the exercise therapy interventions in both groups include spinal exercises, equipment-based strength and endurance training, soccer and table tennis. The BDI-II is administered to both groups to assess the severity of depressive symptoms. Cardiovascular fitness of IG is monitored using a submaximal cycle ergometer aerobic fitness test (PWC test) at baseline and at the end of treatment.

**Results:**

The total number of patients included in the analysis was n=37 in IG (male=78%, M age=42.05) and n=20 in CG (male=60%, M age=47,35). A significant reduction in the BDI-II score was observed in both the IG (M ΔBDI-II=-16.0, p<.001) and CG (M ΔBDI-II=-8.0, p<.05) from the beginning to the end of the treatment period. When comparing reduction in depressive symptoms between two groups, IG achieved statistically significant greater reduction in depressive symptoms compared to the CG (M ΔBDI-II=-8.0, p<.01). Participants in the IG showed a statistically significant improvement in performance, with an increase of 7 watts for the PWC130 and 12 watts for the PWC150 (p<.05), as measured by the submaximal cycle ergometer aerobic fitness test throughout treatment.

**Conclusions:**

The integration of exercise therapy into a multimodal treatment framework was observed to positively impact both physical and mental health outcomes. Given the presence of physical comorbidities and relapses, further research should investigate the long-term impact of behavioral changes related to physically active lifestyle.

**Disclosure of Interest:**

None Declared

